# Comparison of molecular tests for the diagnosis of malaria in Honduras

**DOI:** 10.1186/1475-2875-11-119

**Published:** 2012-04-18

**Authors:** Gustavo A Fontecha, Meisy Mendoza, Engels Banegas, Mitra Poorak, Alexandre M De Oliveira, Tamara Mancero, Venkatachalam Udhayakumar, Naomi W Lucchi, Rosa E Mejia

**Affiliations:** 1 MEIZ-Microbiology School, National Autonomous University of Honduras (UNAH), Tegucigalpa, Honduras; 2 National Malaria Laboratory, Health Ministry, Tegucigalpa, Honduras; 3 Malaria Branch, Division of Parasitic Diseases and Malaria, Center for Global Health, Centers for Disease Control and Prevention, 1600 Clifton Road, Atlanta, GA, 30333, USA; 4 Atlanta Research and Education Foundation, Decatur, GA, USA; 5 Pan American Health Organization (PAHO), Tegucigalpa, Honduras

## Abstract

**Background:**

Honduras is a tropical country with more than 70% of its population living at risk of being infected with either *Plasmodium vivax* or *Plasmodium falciparum*. Laboratory diagnosis is a very important factor for adequate treatment and management of malaria. In Honduras, malaria is diagnosed by both, microscopy and rapid diagnostic tests and to date, no molecular methods have been implemented for routine diagnosis. However, since mixed infections, and asymptomatic and low-parasitaemic cases are difficult to detect by light microscopy alone, identifying appropriate molecular tools for diagnostic applications in Honduras deserves further study. The present study investigated the utility of different molecular tests for the diagnosis of malaria in Honduras.

**Methods:**

A total of 138 blood samples collected as part of a clinical trial to assess the efficacy of chloroquine were used: 69 microscopically confirmed *P. falciparum* positive samples obtained on the day of enrolment and 69 follow-up samples obtained 28 days after chloroquine treatment and shown to be malaria negative by microscopy. Sensitivity and specificity of microscopy was compared to an 18 s ribosomal RNA gene-based nested PCR, two single-PCR reactions designed to detect *Plasmodium falciparum* infections, one single-PCR to detect *Plasmodium vivax* infections, and one multiplex one-step PCR reaction to detect both parasite species.

**Results:**

Of the 69 microscopically positive *P. falciparum* samples, 68 were confirmed to be *P. falciparum*-positive by two of the molecular tests used. The one sample not detected as *P. falciparum* by any of the molecular tests was shown to be *P. vivax*-positive by a reference molecular test indicating a misdiagnosis by microscopy. The reference molecular test detected five cases of *P. vivax/P. falciparum* mixed infections, which were not recognized by microscopy as mixed infections. Only two of these mixed infections were recognized by a multiplex test while a *P. vivax*-specific polymerase chain reaction (PCR) detected three of them. In addition, one of the day 28 samples, previously determined to be malaria negative by microscopy, was shown to be *P. vivax*-positive by three of the molecular tests specific for this parasite.

**Conclusions:**

Molecular tests are valuable tools for the confirmation of *Plasmodium* species and in detecting mixed infections in malaria endemic regions.

## Background

Honduras is a tropical country with more than 70% of its population living at risk of malaria infections. Although malaria control efforts have led to substantial reduction in the number of malaria cases in Central America, low-level transmission of both *Plasmodium vivax* and *Plasmodium falciparum* continues to occur. In Honduras, *P. vivax* is responsible for the largest number of malaria cases while *P. falciparum* accounts for 10-15% of the cases reported [1] and causes a more severe clinical presentation. About 8,000 to 9,000 malaria cases are reported annually, with some fluctuations in the last two years. Microscopy is the most commonly used method of malaria diagnosis. Recently, rapid diagnostic tests (RDTs) were introduced, but their use is currently limited to surveillance studies except in very remote areas where they are used for routine clinical diagnosis with a mandatory microscopic confirmation.

Microscopic detection of malaria parasites is a standard method for the diagnosis of malaria because of its sensitivity, specificity, and ability to quantify the parasitaemia level [2,3] under ideal conditions. It is also less expensive than many other malaria diagnostic tools. In Honduras, microscopy has remained a commonly used diagnostic method for malaria. However, one of the limitations of microscopy is that sometimes it may be difficult to identify the species correctly, especially when slides are not properly prepared or the user has limited training. Microscopy can also fail to detect mixed infections especially when one of the infecting species is present at low levels. Therefore, molecular tests have been used as complementary tools for the diagnosis of malaria in some reference laboratories so that accurate diagnosis can be made. Recent efforts to eliminate malaria, in low-transmission areas such as Central America, have increased the need for introducing molecular tools with high sensitivity capable of detecting sub-clinical levels of parasitaemia in asymptomatic carriers. In recent years, several different polymerase chain reactions (PCR)-based malaria diagnostic methods have been developed [4-9]. Many of these methods have shown ability to detect mixed infections and infections with low parasitaemia [10-12], and most of these methods have been found to be more sensitive than microscopy [11,13].

In this study, the utility of five different molecular tests were investigated for the retrospective detection of 138 microscopically diagnosed samples obtained from a clinical study conducted in Honduras. A commonly used nested PCR test based on the amplification of 18 S ribosomal RNA gene was used as reference test [14]. Other tests included a multiplex PCR for the detection of both *P. falciparum* and *P. vivax* parasites and three single-tube species-specific (two *P. falciparum-*specific and a *P. vivax-*specific test) PCR tests. All these tests, except the nested PCR, were recently developed using novel genome sequences of *P. falciparum* and *P. vivax* as previously reported [15].

## Methods

### Sample collection

A total of 138 blood samples (about 50 μl each) were collected and stored on Whatman filter paper number 3 for molecular testing. These samples were collected in 2009 in the eastern Honduran region of *Gracias a Dios* as part of a clinical trial to assess the efficacy of chloroquine. Only patients with *P. falciparum* mono-infection, as diagnosed by light microscopy, were included in this study. A total of 69 patients were enrolled. The patients’ blood samples were obtained on the day of enrolment (day 0 samples) and 28 days after chloroquine treatment (day 28 samples). Of note, *P. vivax* infections were not included in this clinical study.

### Ethical approval

The study was approved by the Ethics Committee of the Medical Sciences Faculty of the National University of Honduras (UNAH-IRB 00003070). Informed written consent forms were obtained from each participant.

### Microscopy

Blood smears were stained with 3% Giemsa for 30 minutes at room temperature. Smears were analysed by experienced microscopists from the Honduras National Malaria Programme. The standard method recommended by the World Health Organization (WHO) was carried out in order to estimate the number of circulating parasites per μl of blood. As white blood cell (WBC) count was not available for every patient, concentration of 6,000 leucocytes ml^-1^ were used to estimate parasitaemia levels.

### DNA extraction

The DNA was extracted from dried blood spots using a commercial kit (Qiagen, Valencia, CA, USA) following the manufacturer’s instructions. The DNA was eluted in 200 μl of buffer and stored at −20°C until used.

### Nested PCR

The reference 18 s ribosomal RNA gene-base nested PCR was performed with primers and cycling conditions as described by Singh *et al*[16] with some modifications. Briefly, reactions were performed in 25 μL total volume containing 1X buffer, 2.5 mM MgCl_2_, 200 μM dNTPs, 200 nM primers, and 1.25 units of Taq Polymerase (New England Biolabs, Ipswich, MA, USA), and 1-3 μl of DNA template. The genus-specific PCR was followed by *P. falciparum* and *P. vivax* species-specific PCR amplification. Negative and positive controls were run in each reaction. All PCR assays were amplified on a BioRad iCycler (BioRad, Hercules, California). Amplicons from the second PCR were separated by electrophoresis on a 2% agarose gel and stained with ethidium bromide for visualization using ultraviolet trans-illumination. The presence of parasitaemia was confirmed when the expected band size corresponding to *P. falciparum* and *P. vivax* were present.

### Un-nested multiplex and single-tube species-specific PCRs

Various primers used for the different tests are provided in Table 1 and were based on a previous study [15]. Test 1 (primers AL7178/AL7142) and Test 2 (primers AL7140/AL7141) were single tube un-nested PCR tests specific to *P. falciparum*. Test 3 was a multiplex un-nested PCR to detect both *P. falciparum* and *P. vivax* using specific primers (AL7178/AL7142 and AL7175/AL7074, respectively). Test 4 was an un-nested *P. vivax* specific test (primers AL7175/AL7074). Amplifications were performed under the following amplification conditions in a 25 μl volume: 1X Taq Buffer (New England Biolabs, Ipswich MA, USA), 4 mM MgCl_2_, 400 μM each dNTP, 500 nM each primer, 2.5 units of Taq DNA Polymerase (New England Biolabs, Ipswich, MA, USA), and 1-3 μl of DNA template. Reactions were amplified by an initial denaturation at 95°C for 2 min, 35 cycles of 95°C for 30 sec, annealing temperature for 30 sec, and 72°C for 45 sec, with a final extension at 72°C for 5 min. Amplicons were visualized by 2% agarose gel electrophoresis with ethidium bromide. Each molecular test was performed two times. If a discordant result was obtained, the experiment was repeated a third time and the final result was determined by two concordant tests.

**Table 1:** Primers and PCR conditions used for the different molecular tests evaluated

**Test (species)**	**Primer ID**	**Primer sequence 5′-3′**	**Annealing temperature**	**Size (bp)**
Test 1 (Pf)*	AL7178	CCGGAAATTCGGGTTTTAGAC	60°C	220
	AL7142	GCTTTGAAGTGCATGTGAATTGTGCAC		
**Test 2 (Pf)**	AL7140	CCATTTTACTCGCAATAACGCTGCAT	57°C	716
	AL7141	CTGAGTCGAATGAACTAGTCGCTAC		
**Test 3 (Pf/Pv)**	AL7178 AL7142	CCGGAAATTCGGGTTTTAGAC GCTTTGAAGTGCATGTGAATTGTGCAC	60°C	333/220
	AL7175	CTGATTTTCCGCGTAACAATG		
	AL7074	CAAATGTAGCATAAAAATCYAAG		
Test 4 (Pv)*	AL7175	CTGATTTTCCGCGTAACAATG	54°C	333
	AL7074	CAAATGTAGCATAAAAATCYAAG		

Table 1 shows the primer ID and sequence and PCR conditions used for the novel molecular test used to detect *Plasmodium falciparum* (Pf) and/or *Plasmodium vivax* (Pv). *Primers used in the multiplex Test 3

## Results and conclusions

Molecular tests for malaria diagnosis have repeatedly been shown to be more sensitive and accurate in detecting malaria parasites compared to microscopy [17,18]. In this study, 69 microscopically confirmed *P. falciparum*-positive samples and 69 samples obtained after day 28 of chloroquine treatment (microscopically found to be negative for malaria parasites) were used to test the utility of four novel molecular tests for malaria diagnosis. The results of these different tests were compared to the reference nested PCR test (Table 2). Of the 69 microscopically positive *P. falciparum* samples used in this study (parasite count range 320–120,000 parasites/μl), 68 were confirmed to be *P. falciparum*-positive by the reference nested PCR test and Test 1. One sample (PL3333), which was found to be *P. falciparum* by microscopic examination, was not confirmed to be positive by any of the *P. falciparum* molecular tests used. Interestingly, the same sample was found to be *P. vivax*-positive by the reference nested PCR test and two other molecular *P. vivax* tests used (Test 3 and Test 4). This finding suggests that this sample was misdiagnosed as *P. falciparum* by microscopy.

**Table 2:** **Sensitivity and specificity of the novel primers to detect*Plasmodium falciparum*compared to the reference nested PCR**

**Reference test**	**Test 1**		**Test 2**		**Test 3**	
**Nested PCR**	**AL7178/AL7142 (Pf)**		**AL7140/AL7141 (Pf)**		**Multiplex (Pf/Pv)**	
	Positive	Negative	Positive	Negative	Positive	Negative
Positive (68)	68	0	63	5	66	2
Negative (68)	0	68	0	68	0	68
Sensitivity	100% (CI: 93.4-100%)		92.6% (CI: 83.0-97.3)		97.1% (CI: 88.8-99.5)	
Specificity	100% (CI: 93.4-100)		100% (CI: 93.4-100)		100% (CI: 93.4-100)	

The nested PCR test was used as a reference test. Test 1 and Test 2 are specific to *Plasmodium falciparum* (Pf). Test 3 is a multiplex test designed to detect both *Plasmodium falciparum* and *Plasmodium vivax* (Pv). 68 parasite negative samples were identified as negative by all the tests

Interestingly, the reference nested PCR test detected the presence of *P. vivax/P. falciparum* mixed infections in five of the samples previously identified as *P. falciparum* mono-infections using microscopy. Previous studies have reported on the fact that mixed infections are often not recognized or are underreported [19-22], mainly due to the limitation of detection tools employed [22]. The current study confirms these previous studies and demonstrates that low-level mixed infections are indeed common and molecular tools are needed to detect them. In addition, one of the day 28 samples, previously diagnosed as malaria negative by microscopy, was clearly identified to be *P. vivax* using the reference nested PCR test. This finding was confirmed by the other tests designed to detect *P. vivax* (Test 3 and Test 4). This finding again highlights the importance of using complementary molecular tests for species confirmation as needed.

The performance of Test 1 was comparable to that of the reference nested test in detecting all *P. falciparum* infections (Table 2). On the other hand, Test 2 showed lower sensitivity as compared to both the reference nested PCR test and Test 1. This finding suggests that Test 1 is a better alternative to the reference nested PCR test for detecting *P. falciparum* as it does not require two rounds of PCR, which often leads to risks of contamination and is more expensive and time consuming [11]. Since Test 2 showed the lowest sensitivity for *P. falciparum* detection, it may not be a good complementary test unless its sensitivity can be improved with further alterations.

The multiplex Test 3 was 97.1% sensitive in detecting *P. falciparum* infection compared to the reference nested PCR test (Table 2). This test also detected two of the five *P. vivax* mixed infections detected by the reference nested PCR test. Although the multiplex Test 3 needs further improvement to increase its sensitivity, it has performed better than previously described nested multiplex PCR tests [8,23].

Test 4, which was a *P. vivax*-specific test, detected four of the six *P. vivax*-positive samples detected by the reference nested PCR test. Sensitivity and specificity of this test were not calculated since the number of *P. vivax* samples was too low to undertake this evaluation. Five of these samples were shown to be mixed infections with *P. falciparum* and the other one was shown to be a *P. vivax* sample. The fact that these *P .vivax*/*P. falciparum* mixed samples were not detected by microscopy as mixed infections to begin with, may indicate that the *P. vivax* parasitaemia may be too low in these samples and therefore, below the detection limit of this test. It is known that the density of *P. vivax* infection in general is low compared to *P. falciparum* and, consequently, a highly sensitive PCR test similar to the reference nested PCR test will be required to detect mixed infections correctly. Although further optimization is required to improve the sensitivity of Test 4, it is noteworthy that this test was able to detect four of the *P. vivax* samples that microscopy did not detect.

As illustrated by results from this study, one of the most notable advantages of molecular methods is their higher sensitivity to detect mixed infections and to identify species of malaria parasites accurately. Therefore, while light microscopy is still a convenient technique for the routine malaria diagnosis in countries like Honduras, molecular tests are suitable complementary tests for the confirmation of species and to detect mixed infections in special studies such as drug efficacy clinical trials.

## Competing interests

The authors declare that they have no competing interests.

## Authors’ contributions

GF and MM carried out the DNA extraction and GF, MM, MP and NL performed molecular experiments. GF, NL and VU drafted the manuscript and other authors read and edited the manuscript. RM and EB participated in sample collection and microscopic analyses coordination. TM, GF, NL, AMO, RM, and VU conceived the study and participated in its design and coordination and helped to draft the manuscript. All authors read and approved the final manuscript.
